# The Late Epidemics in Louisiana and Mississippi

**Published:** 1898-11

**Authors:** 


### The Late Epidemics in Louisiana and Mississippi.

Those of the older physicians whose experience with yellow fever was acquired in the memorable epidemics of long ago—say 1867-'73-'78—are somewhat at sea with regard to the new issue; the so-called yellow fever of 1897-8. If it is yellow fever, it has falsified its record in many respects; it no longer answers to the roll call of characteristics, and its "mildness,"—want of virulence; its small mortality, are unaccountable. It was never known in former years to strike a communinty and let up in hot weather, so long as there was material to feed upon. Of this last departure Galveston and Houston, (October, 1897), can be cited as furnishing an instance. If it was yellow fever, it is strange that it "quit" in October, while it was still hot. (Or else,—and it is hardly to be supposed, the doctors at Galveston who believed it was yellow fever, failed to report cases after about October 20.) That it either "quit" or was no longer reported,—I cite in testimony the letter of Dr. Guiteras to Surgeon General Wyman, of the M. H. S. Under date of October 26, 1897, Dr. Guiteras wrote as follows:

"SIR:—The fact that no cases of yellow fever have been reported by the Boards of Health of Galveston and Houston since my report on several cases there found, is a matter of serious concern.

"I deem it my duty to state that in my opinion, it is not possible for the yellow fever to have disappeared from those cities at the present time. The disease was already spreading and beyond the control of the means usually employed for its suppression.

"The neighboring cities should be warned against trusting in the apparent arrest of the disease.

"Very respectfully, your obedient servant,

"JOHN GUITERAS,

"A. A. Surgeon, M. H. S."

So, Dr. Guiteras—an old time yellow fever doctor—having diagnosed the disease in Galveston and Houston to be yellow fever, confidently expected it to spread and to continue till frost. It didn't do so.

In Franklin, La.—out of something like three hundred and sixty "cases" reported (to October 15),—only six died. At Clinton and Wilson, La., the mortality of the fever that made its appearance there and was called yellow fever—and accordingly was quarantined—was about 2 per cent. At Ocean Springs, Miss., last year, where the disease first made its appearance, it was said and generally credited, that four hundred cases of dengue had been treated there before a death occurred; when—all of a sudden—a death took place—from black vomit. Then the disease became yellow fever. From Ocean Springs the infection spread to the interior of Mississippi and to New Orleans and other points in Louisiana. At Edwards, Miss., where the infection was carried in the trunks of Edwards people who had been summering at Ocean Springs—an epidemic of yellow fever at once set up—Dr. Champion, a local physician, being the first victim; and the mortality was something like the old time yellow fever—pretty heavy—and the disease raged till after frost—extending out into the adjacent country—like the old time epidemics of '67, '73 and '78. At New Orleans this year—up to October 15 (ult.), there had been 74 cases reported to and by the State Board of Health, with 19 deaths; 25.6 per cent. That was like old times,—the mortality there in '78 being, according to the late Prof. Bemis, (Ref. H. Bk. Med. Sci.), 16.66. But in reregard to New Orleans it may be said that all the cases of yellow fever and supposed yellow fever were not reported to the Board—because—the doctors there are divided in opinion as to the disease—as they are elsewhere—many claiming that it is not yellow fever, but *aggravated dengue;* and who like the writer, do not subscribe to any "mild form" of yellow fever, any more than they will admit that there is a mild kind of strychnia or hemlock. Mild cases—yes, because some persons get less poison than others; some have greater powers of resistance; but that there can be a "mild epidemic" of any virulent disease is a paradox. Yellow fever is yellow fever, and is the same everywhere, where there are subjects.

Whatever the disease is, whether yellow fever or dengue, and no doubt many cases of dengue have passed for yellow fever—it seems to have swept over a large part of the South pretty generally. We had in San Antonio and in Austin and other towns last year the same disease, in my opinion, that they had at Galveston and Houston, and this year at Baton Rouge, Franklin, Wilson and Clinton, La., and which disease—occurring elsewhere would probably have been called yellow fever. It will be observed that as a rule, towns visited last year with this fever have escaped this year. It is open to serious question whether there has been any yellow fever at Tampico this year; the disease there, called yellow fever, was quite fatal to new comers and the lower classes, but it disappeared spontaneously in October, and the local physicians deny that it was yellow fever. This fever has spread now—November 1,— away up into the mountains of Mexico, and is now raging at Monterey and, (it is reported, even at Saltillo), at an altitude which has heretofore been thought too great for yellow fever. There, as elsewhere, the "doctors differ." Dr. Turpin, the Texas quarantine officer at Laredo, Texas, having been officially directed to go to Monterey and examine and report on the disease, unhesitatingly pronounced it yellow fever, and quite fatal. Dr. Cross, of Monterey, late of San Antonio, also so pronounced it. *Per contra,* Dr. R. H. L. Bibb, of Saltillo,—chief surgeon of the Mexican National Railroad, who is an old yellow fever doctor, well known, perhaps, to every Texas reader of this as an old, popular and successful Texas physician, went to Monterey and investigated the disease. He wired the State Health Officer in October that he had examined forty cases, in all stages, and that it was unquestionably dengue; that a local physician there had treated five hundred cases without a death. All of a sudden a case dies of black vomit. What is it? Dengue seems to merge into (?) yellow fever, an impossibility; it is one or the other; which is it? If it is yellow fever, why is it so "mild" as to be mistaken for dengue and only recognized when it ceases to be mild? Why fatal in some towns and harmless in others?

The problem of yellow fever is not yet solved. That there is a kinship between yellow fever and dengue there is much reason to believe; but what is it? What relation do they bear to each other? Until the bacillus (by which the yellow fever is generally believed to be caused) is demonstrated and cultivated,—and it will be some day—the problem will not be solved. I do not believe there is a man on earth who can differentiate dengue and yellow fever when both are prevailing.

We reproduce in this issue a strong paper favoring the view that there was some yellow fever at Galveston last year. The paper is by Dr. H. A. West, late Professor of Practice in the Texas Medical College, and was read at the meeting of the Texas Medical Association at Houston in April last. Our apology for devoting so much space to the subject, if any be needed, is that the subject is now uppermost in the minds of Southern physicians, who realize that truly very little is yet known of the disease. Dr. West makes out a strong case in favor of yellow fever. Dr. C. H. Wilkinson, of Galveston, also, who practiced there in the great epidemic of 1867, contemporary with the writer of this,—takes a different view from that of Dr. West, and in an article published last winter makes out an equally strong showing that the fever was dengue; and so it goes; doctors still differing.

In this connection we ask our readers to refer to and read the letter of Dr. G. S. West, of Palestine, Texas, to the editor of this journal,—published in our May, 1898, issue. Dr. West has seen and treated more yellow fever perhaps than any man in Texas; his experience goes back to Norfolk in the memorable scourge of 1850-1853. Dr. West unqualifiedly denies that there was a single case of yellow fever in Galveston or Houston last fall; and I think I may say that before he died Dr. Swearingen had about been convinced that such was the case. So, if the disease be really dengue, and, outside of exceptional places it has been less fatal than *grippe*, sanitary science has a new problem to grapple with, and a new "quarantinable disease" (?) has been added to the list.

